# MicroRNA Expression Signature in Degenerative Aortic Stenosis

**DOI:** 10.1155/2016/4682172

**Published:** 2016-08-04

**Authors:** Jing Shi, Hui Liu, Hui Wang, Xiangqing Kong

**Affiliations:** Department of Cardiology, The First Affiliated Hospital of Nanjing Medical University, Nanjing 210029, China

## Abstract

Degenerative aortic stenosis, characterized by narrowing of the exit of the left ventricle of the heart, has become the most common valvular heart disease in the elderly. The aim of this study was to investigate the microRNA (miRNA) signature in degenerative AS. Through microarray analysis, we identified the miRNA expression signature in the tissue samples from healthy individuals (*n* = 4) and patients with degenerative AS (*n* = 4). Six miRNAs (hsa-miR-193a-3p, hsa-miR-29b-1-5p, hsa-miR-505-5p, hsa-miR-194-5p, hsa-miR-99b-3p, and hsa-miR-200b-3p) were overexpressed and 14 (hsa-miR-3663-3p, hsa-miR-513a-5p, hsa-miR-146b-5p, hsa-miR-1972, hsa-miR-718, hsa-miR-3138, hsa-miR-21-5p, hsa-miR-630, hsa-miR-575, hsa-miR-301a-3p, hsa-miR-636, hsa-miR-34a-3p, hsa-miR-21-3p, and hsa-miR-516a-5p) were downregulated in aortic tissue from AS patients. GeneSpring 13.1 was used to identify potential human miRNA target genes by comparing a 3-way comparison of predictions from TargetScan, PITA, and microRNAorg databases. Gene Ontology (GO) and Kyoto Encyclopedia of Genes and Genomes (KEGG) analysis were performed to identify potential pathways and functional annotations associated with AS. Twenty miRNAs were significantly differentially expressed between patients with AS samples and normal controls and identified potential miRNA targets and molecular pathways associated with this morbidity. This study describes the miRNA expression signature in degenerative AS and provides an improved understanding of the molecular pathobiology of this disease.

## 1. Introduction

Degenerative aortic stenosis (AS), the most common valvular heart disease in the elderly, has become a leading cause for surgical valve replacement in industrialized countries. As a result of rising life expectancy and ageing populations, the global prevalence of AS is increasing and is expected to surpass 10 billion by 2100 [1–3].

Degenerative AS has long been considered a chronic process with gradual deposition of calcium phosphate in the valve occurring with age. However, emerging evidence has indicated that this condition is mediated by the interplay of complex biological processes that include the following: inflammation, cell apoptosis, lipids deposition, renin-angiotensin system activation, remodeling of the extracellular matrix, and bone formation [4–11]. However, the underlying mechanisms that regulate this process remain largely unknown [12, 13].

MicroRNAs (miRNAs) are small, 21–25-nucleotide, endogenous, single-stranded noncoding RNAs that regulate target gene expression by binding messenger RNAs (mRNAs) and inhibiting or reducing translation. A single miRNA can regulate numerous genes, while a single gene can be regulated by multiple miRNAs [14]. miRNAs play a critical role in many physiological processes, and there is a growing body of studies indicating that distinct patterns of altered miRNA expression are associated with specific disease processes [15–19]. In the present study, we explore the miRNA expression signature of degenerative AS to improve our understanding of the molecular alternations in this disease.

## 2. Methods 

### 2.1. Tissue Samples Collection and RNA Isolation

Written informed consent was obtained from all participants of age and/or via their parents. All procedures in this study were approved by the Ethics Committees of the First Affiliated Hospital of Nanjing Medical University and conformed to the principles outlined in the Declaration of Helsinki. Tissue samples from four healthy controls were obtained from prospective multiorgan donors without cardiovascular pathology in cases in which technical reasons prevented transplantation. Aortic valves from patients with degenerative AS were obtained from four patients who underwent surgical valve replacement. General characteristics of these participants are displayed in Table 1. Gross and histological examination was performed to confirm the presence/absence of AS in each sample. Valve samples were immediately snap-frozen in liquid nitrogen upon collection. Total RNA was extracted using an RNeasy Mini Kit in accordance with the manufacturer's protocol (Qiagen, Hilden, Germany).

### 2.2. RNA Labeling and Array Hybridization

miRNA expression was evaluated using Agilent miRNA arrays (V2), which included 723 human and 76 human viral miRNAs from the Sanger database v.10.1 (Agilent Technologies, Foster City, CA). Total RNA was dephosphorylated and ligated with pCp-Cy3 and subsequently hybridized to the arrays. After samples were washed and scanned, using Agilent Scan Control software, the Agilent Feature Extract software v9.5.3 was used to analyze the arrays. Each sample was evaluated in triplicate. miRNA expression data were normalized using a bead-based assay and the locally weighted smooth spline (LOWESS) method. After normalization, all expression values were transformed to a linear scale for statistical analysis.

### 2.3. Bioinformatic Analysis

GeneSpring 13.1 was used to identify potential human miRNA target genes, which compared TargetScan, PITA, and microRNAorg databases and created a Venn diagram to demonstrate relations among the databases. Gene Ontology (GO) analysis was performed to investigate the biological processes, cellular components, and specific molecular function of differentially expressed coding genes identified. Pathway analysis was used to determine the involvement of coexpressed genes in different biological pathways according to Kyoto Encyclopedia of Genes and Genomes (KEGG).

### 2.4. Statistical Analysis

Independent Student's *t*-test was used to determine whether there were any significant differences between the miRNA expression profiles between two groups. *p* values less than 0.05 (*p* < 0.05) were considered to be statistically significant. Significant data were further analyzed by cluster analysis, and the expression profiles were visualized with GeneSpring 10.0 (Agilent Technologies).

## 3. Results

### 3.1. Unsupervised Hierarchical Cluster Analysis of miRNA Microarray Data

**Figure 1:**
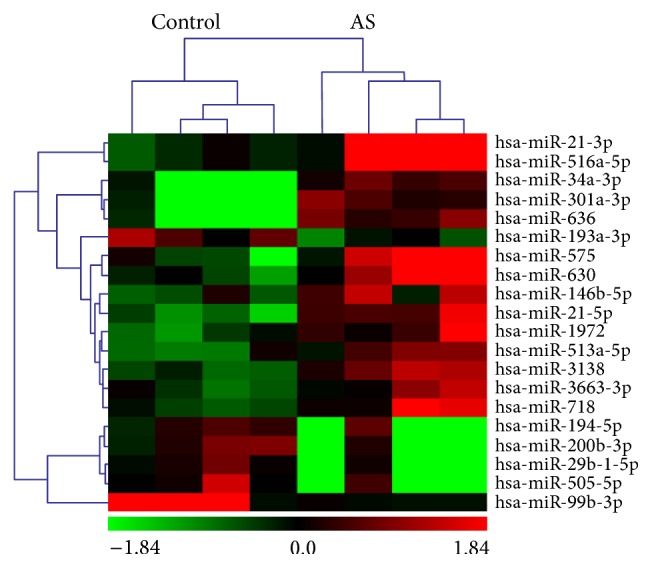
Unsupervised hierarchical clustering identified two distinct groups (control versus AS) based on their miRNA expression profile. Sample names are listed at the top. The names of the significantly (*p* < 0.05) differentially expressed miRNAs are shown on the right. Twenty miRNAs were expressed differently in target genes analysis.

miRNA microarray identified 20 miRNAs with significantly differential expression (>2.0-fold) in AS samples relative to normal controls. Six miRNAs were overexpressed (hsa-miR-193a-3p, hsa-miR-29b-1-5p, hsa-miR-505-5p, hsa-miR-194-5p, hsa-miR-99b-3p, and hsa-miR-200b-3p) and 14 (hsa-miR-3663-3p, hsa-miR-513a-5p, hsa-miR-146b-5p, hsa-miR-1972, hsa-miR-718, hsa-miR-3138, hsa-miR-21-5p, hsa-miR-630, hsa-miR-575, hsa-miR-301a-3p, hsa-miR-636, hsa-miR-34a-3p, hsa-miR-21-3p, and hsa-miR-516a-5p) were downregulated in aortic tissue from AS patients (Table 2). Unsupervised hierarchic clustering was performed based on the 20 differentially expressed miRNAs and displayed as heat map (Figure 1).

**Figure 2:**
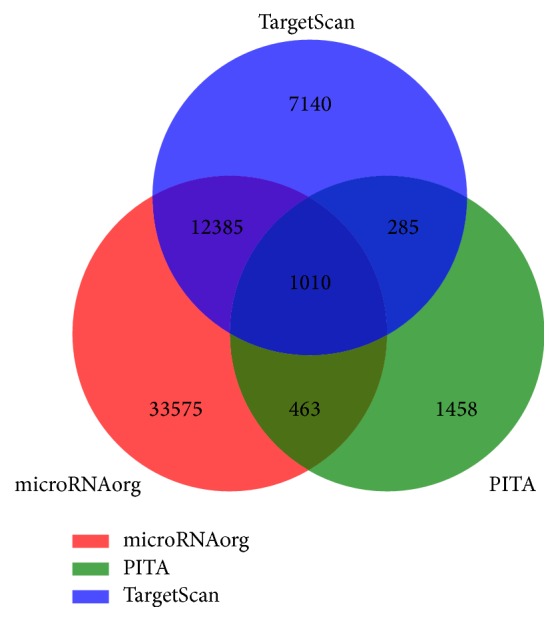
The red, green, and blue sets stand for target genes predicted by databases microRNAorg, TargetScan, and PITA, respectively.

microRNAorg, TargetScan, and PITA were used to predict the targets of differentially expressed miRNAs in CAVD samples using software GeneSpring 13.1. A Venn diagram was generated to highlight the relations among the 3 databases. There are 1010 overlapping genes identified by all 3 programs, which are most likely to be targets of miRNAs in patients with AS (Figure 2).

### 3.2. GO and Pathway Analysis

Gene Ontology (http://geneontology.org/) was used to classify the function of up- and downregulated genes from 3 structured networks: biological processes, cellular components, and molecular function.

**Figure 3:**
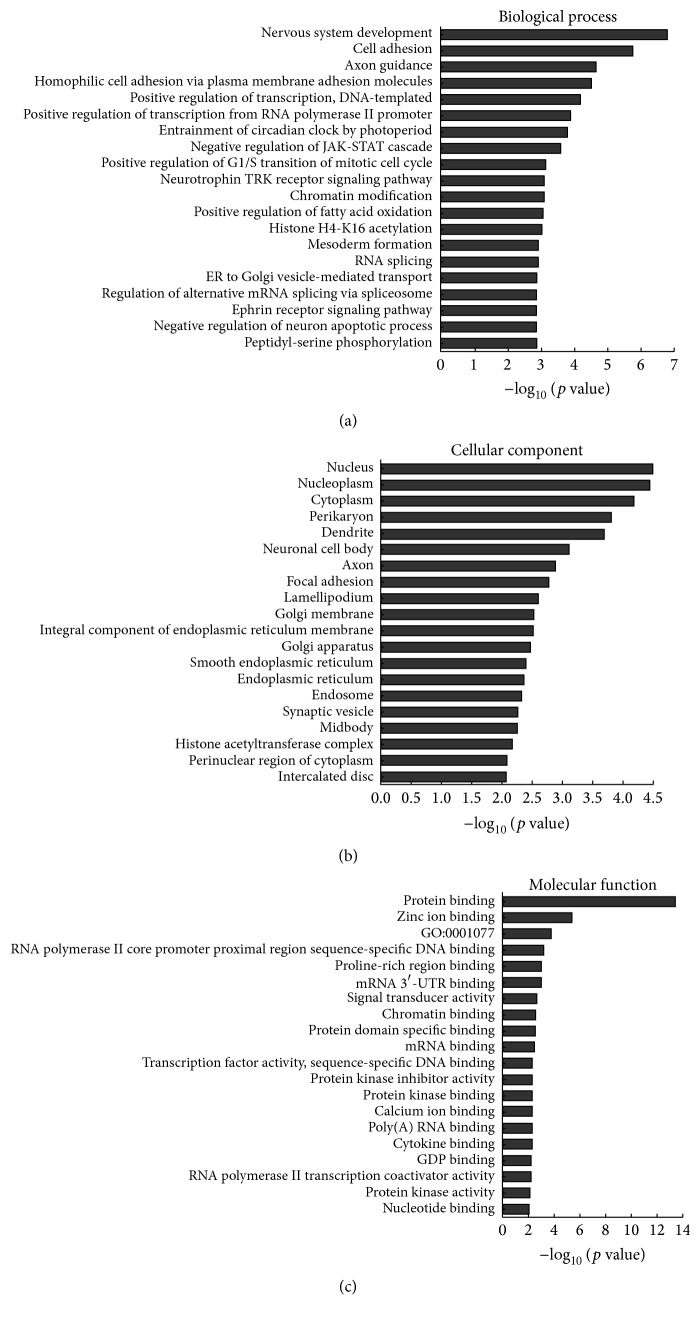
GO analysis for differentially expressed mRNAs. (a)–(c) GO analysis according to biological process, cellular component, and molecular function, respectively, ranked by enrichment score (−log_10_ (*p* value)).

**Figure 4:**
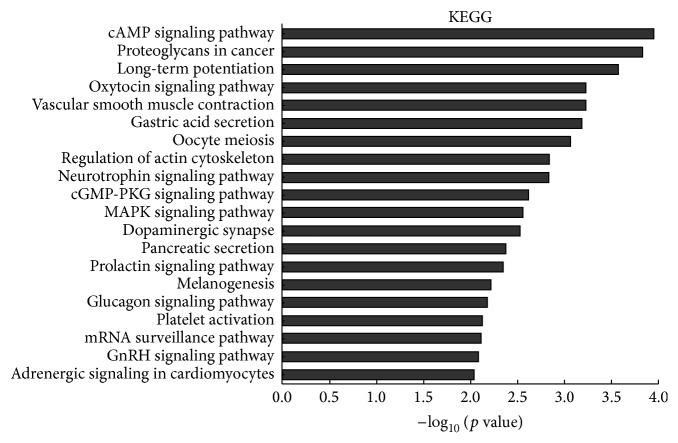
Pathway analysis based on the KEGG database. Ranked by enrichment score (−log_10_ (*p* value)).

In this study, differentially expressed mRNAs were enriched in numerous biological processes including the following: cell adhesion, homophilic cell adhesion, positive regulation of transcription, negative regulation of JAK-STAT cascade, and positive regulation of G1/S transition of mitotic cell cycle associated with biological processes (Figure 3(a)). Similarly, the following cellular components were affected: nucleus, nucleoplasm, and cytoplasm linked with cellular components (Figure 3(b)), while affected molecular functions include the following: protein binding, zinc ion binding, and transcriptional activator activity involved in molecular functions (Figure 3(c)). Moreover, KEGG pathway analysis identified significantly (*p* < 0.05) affected pathways including the following: cAMP signaling pathway, vascular smooth muscle contraction, regulation of actin cytoskeleton, neurotrophin signaling pathway, and cGMP-PKG signaling pathway (Figure 4).

## 4. Discussion

Recent studies have improved our understanding of the mechanisms underlying AS [12, 20]. However, there are not many reports that have investigated the function of miRNAs as they relate to the pathobiology of AS [21–26].

A microRNA expression signature provides a better understanding of the mechanisms of a disease [27]. In the present study, we explored miRNA expression signatures associated with degenerative AS using miRNA microarray analysis. Six overexpressed miRNAs (hsa-miR-193a-3p, hsa-miR-29b-1-5p, hsa-miR-505-5p, hsa-miR-194-5p, hsa-miR-99b-3p, and hsa-miR-200b-3p) and 14 downregulated miRNAs (hsa-miR-3663-3p, hsa-miR-513a-5p, hsa-miR-146b-5p, hsa-miR-1972, hsa-miR-718, hsa-miR-3138, hsa-miR-21-5p, hsa-miR-630, hsa-miR-575, hsa-miR-301a-3p, hsa-miR-636, hsa-miR-34a-3p, hsa-miR-21-3p, and hsa-miR-516a-5p) were identified in patients with AS, relative to normal controls, and their general characteristics and functional annotations were analyzed using bioinformatic tools.

There were other miRNA microarrays performed about AS before, and, in these studies, the main cause of AS is calcification of the aortic valves [20, 22]. However, as rheumatic fever remains to be the most important etiological factor in calcific aortic valve disease (CAVD) in China, we do not choose CAVD patients to reduce confounding factors when exploring the mechanisms that underlie degenerative AS.

As a limitation of our study, the specific pathways affected by miRNAs and which cause AS remain elusive. Further studies are required to functionally characterize the role of specific candidate miRNAs.

In conclusion, the present study improved our understanding of the role of miRNAs in degenerative AS. Our findings provide improved understanding of the molecular alterations in this disease and may provide potential targets for future clinical applications.

## Figures and Tables

**Table 1:** Characteristics of the participants.

	Control	AS	*p* value
Number	4	4	ns
Male	4	4	ns
Age	41.5 ± 10.4	58.3 ± 4.6	0.0429
LVEF < 50%	0	0	ns
Coronary disease	0	0	ns
Diabetes mellitus	0	0	ns
Medications	0	0	ns

Values are displayed as mean ± standard deviation.

LVEF: left ventricular ejection fraction.

**Table 2:** Six overexpressed and 14 downregulated miRNAs in aortic tissue from AS patients compared to the control group.

Systematic name	*p* value	FC (abs)	Regulation
hsa-miR-193a-3p	0.020637	2.067236	Up
hsa-miR-29b-1-5p	0.0222	11.56757	Up
hsa-miR-505-5p	0.030211	13.22606	Up
hsa-miR-194-5p	0.036807	14.95685	Up
hsa-miR-99b-3p	0.029772	15.98001	Up
hsa-miR-200b-3p	0.021638	20.1747	Up
hsa-miR-3663-3p	0.042986	2.063909	Down
hsa-miR-513a-5p	0.01703	2.238355	Down
hsa-miR-146b-5p	0.040094	2.244855	Down
hsa-miR-1972	0.033015	2.432029	Down
hsa-miR-718	0.033461	2.559791	Down
hsa-miR-3138	0.002648	2.69498	Down
hsa-miR-21-5p	0.004216	3.334316	Down
hsa-miR-630	0.020492	3.564175	Down
hsa-miR-575	0.029949	5.137385	Down
hsa-miR-301a-3p	0.011849	20.77046	Down
hsa-miR-636	0.010177	25.1902	Down
hsa-miR-34a-3p	0.013993	25.44595	Down
hsa-miR-21-3p	0.02304	32.11932	Down
hsa-miR-516a-5p	0.022169	41.76603	Down
